# The Relationship Between Self-Efficacy and Job Satisfaction: A Meta-Analysis from the Perspective of Teacher Mental Health

**DOI:** 10.3390/healthcare13141715

**Published:** 2025-07-16

**Authors:** Yu Xiao, Li Zheng

**Affiliations:** 1 School of Education, Tsinghua University, Beijing 100084, China; xiaoyu921@tsinghua.edu.cn; 2 School of Education, Shanghai Normal University, Shanghai 200234, China

**Keywords:** teacher well-being, mental health, self-efficacy, job satisfaction, meta-analysis

## Abstract

Teacher mental health is a growing concern in educational and public health systems worldwide. This meta-analysis systematically examines the relationship between teacher self-efficacy—a core construct in social cognitive theory—and job satisfaction, both considered crucial indicators of occupational well-being. A total of 39 studies involving teachers across 18 countries were analyzed, yielding a significant positive correlation between self-efficacy and job satisfaction (r = 0.41, *p* < 0.001), with notable heterogeneity (I^2^ = 97%). Subgroup analyses revealed that the relationship was significantly stronger among teachers in high school and post-secondary contexts, and in studies conducted in Southern Hemisphere countries, highlighting the role of contextual and systemic moderators such as educational level and geographic inequality. The findings underscore the need for school- and policy-level interventions that bolster teachers’ self-efficacy through professional development, emotional support, and improved working conditions. Such interventions are essential not only for enhancing job satisfaction and reducing attrition but also for promoting the psychological resilience and well-being of the global teaching workforce. The study provides evidence-based insights into education and health policymakers aiming to support teacher retention and mental health through scalable, targeted initiatives.

## 1. Introduction

Given the increasing recognition of educators’ mental health as a component of occupational health [1], understanding the factors that promote job satisfaction is crucial for sustaining healthy school systems and retaining qualified personnel. In this study, we use the term “mental health and well-being” to refer to a teacher’s overall psychological functioning, including emotional stability, stress resilience, life satisfaction, and sense of purpose at work [2]. This definition aligns with existing frameworks that define mental well-being as both the absence of negative states (e.g., burnout, anxiety) and the presence of positive resources (e.g., motivation, engagement, self-efficacy). Self-efficacy, in particular, is increasingly viewed in the occupational health literature not only as a motivational factor but also as a buffer against stress and burnout [3]. Embedding teacher self-efficacy within this broader framework of occupational mental health allows us to better understand its role in enhancing teachers’ emotional resilience, reducing psychological distress, and improving long-term well-being. This integrative approach also aligns with the goals of health-oriented research that aims to promote both psychosocial and professional sustainability in high-stress professions such as teaching. Teachers’ job satisfaction is a critical factor that influences not only educators themselves, but also student learning and overall school performance, and understanding what drives teacher satisfaction is therefore essential to improving the quality and outcomes of education [4,5,6]. In this way, we need to examine what key factors affect job satisfaction and thus explore what potential measures can be taken to improve job satisfaction. A range of external factors can significantly affect teachers’ job satisfaction, such as salary, promotion, and relationships with colleagues [7,8,9,10]. In addition to external factors, scholars suggested that internal factors including teachers’ self-efficacy, work experience, gender, and grade level taught are significantly associated with job satisfaction. Specifically, higher levels of self-efficacy led to higher job satisfaction among teachers [11,12].

Most of the factors discussed above are difficult or impossible to change through intervention. For example, teacher’s salary, promotion opportunities, relationships with colleagues, teaching experience, gender, and student population taught are all objective conditions that are difficult to change subjectively. Speaking of the internal factors, such as teachers’ work experience, a study indicated a significant difference between experienced and inexperienced teachers in job satisfaction [13], as inexperienced teachers seemed less likely to be satisfied due to their unfamiliarity with their work. In terms of gender, a study found female teachers had greater work–family conflict and work-related stressors, leading to increased risk of burnout and lower job satisfaction as a result of their higher role conflict between work and domestic roles [14]. Differences in teachers’ perceived job satisfaction were revealed by the grade level taught as the most satisfied teachers were high school teachers, while the least satisfied were middle school teachers [15]. Based on the above discussion, self-efficacy can be particularly valuable to transform teachers’ job satisfaction because it is highly manipulable, with Bandura indicating that self-efficacy is composed of different sources of efficacy expectations; changing any one of the sources can shift efficacy perceptions, and each of these sources is alterable [16]. This makes self-efficacy a potentially powerful protective factor against occupational stress and emotional exhaustion in teaching.

The relationship between self-efficacy and job satisfaction warrants further investigation. The results of the existing studies produce contradictory results regarding the possible relationship between teachers’ self-efficacy and teacher job satisfaction. Based on a recent study, teachers with higher self-efficacy tend to be better satisfied with their job as they have a greater likelihood of being inherently driven, positively engaging in course design, and delivering purposeful instructional activities for their students [17]. However, several studies are showing that self-efficacy has no significant impact on job satisfaction (e.g., [18,19,20]). In the field of educational sciences, an independent study cannot be expected to produce definitive results [21]. Whether the relationship between self-efficacy and teacher job satisfaction measured in these studies is consistent and whether the overall effect is significant needs to be studied in more depth. Given the growing interest in supporting teacher resilience, emotional well-being, and retention as part of systemic health policy efforts, further clarification of this relationship is both timely and essential.

This study is unique in that it systematically investigates the moderating factors that influence the relationship between teacher self-efficacy and job satisfaction, this focus allows for a more fine-grained understanding of when and for whom self-efficacy most strongly contributes to job satisfaction. Recent studies reflect notable theoretical and empirical advances in this domain. One study extends the understanding of self-efficacy by embedding it within broader school leadership and social support frameworks, advancing contextual models of teacher well-being [22]. Another study contributes new insights by linking self-efficacy to motivation and job satisfaction through differentiated patterns across teacher demographics, prompting more tailored intervention strategies [23]. A third study enriches the field by situating teacher self-efficacy within under-researched vocational education settings, integrating both external (school climate) and internal (self-beliefs) determinants of satisfaction [24]. These studies represent the most recent theoretical and empirical developments relevant to this meta-analysis. The remainder of this article is organized as follows: Section 2 describes the inclusion criteria and methodological approach; Section 3 presents the results of the meta-analysis; Section 4 discusses the theoretical and practical implications; and Section 5 concludes with limitations and suggestions for future research.

## 2. Literature

### 2.1. Teachers’ Job Satisfaction

Job satisfaction is a complex psychological construct that reflects how individuals evaluate their professional experience and work context [25]. In educational research, this concept is often examined through the lens of social cognitive theory [16], which emphasizes the role of individual agency and belief systems—particularly self-efficacy—in shaping work-related outcomes. Teachers who perceive themselves as competent and effective are more likely to experience positive emotions about their job, sustain motivation, and persist through challenges, all of which contribute to higher job satisfaction [26,27].

Therefore, understanding teachers’ job satisfaction requires integrating historical perspectives with contemporary theories of motivation and cognition. Job satisfaction has been the topic of pioneering and critical research in the social sciences [28]. A critical trigger of this concept is the Hawthorne experiment, which lasted five years from 1927 to 1932. The purpose of the experiment was to show the possible effects of environmental conditions (mostly illumination) on worker productivity. The results showed that employees’ work behavior was influenced by their work mood, thereby further affecting their productivity (known as the Hawthorne Effect). A significant implication of this study is that it demonstrated that people work for more than just salaries. These findings on employees’ psychosocial health factors provide a pavement for future job satisfaction studies [29].

Since the concept’s inception, it has been defined differently by different scholars (e.g., [30,31,32,33,34]). The concept of job satisfaction originated from Fisher and Hanna in 1931 as they first characterized job satisfaction as a product of non-regulatory mood tendency [35]. One early study defined job satisfaction as a subjective response to the work situation. Specifically, job satisfaction is a psycho-physiological perception related to the work environment [30]. Other scholars viewed job satisfaction as the degree to which an individual’s expectations match with what an employee gets [31]. In other words, dissatisfaction arises when expectations are not fulfilled, and job satisfaction appears only when the actual gains from work are greater than the employee’s expectations. Locke defined job satisfaction as a positive emotional and mental state derived from an organization member’s assessment of his or her job or prior experience [31]. Spector recognized job satisfaction as the overall feeling of an employee about his/her job [34]. These provided a macroscopic, occupation-neutral perspective on the concept of job satisfaction, its origins, and its academic research. Technological change can shape employees’ job satisfaction in both positive and negative ways, as it may lead to increased efficiency and role clarity (improvement effect), while also creating anxiety over job displacement (replacement stress), ultimately influencing job satisfaction in complex ways [36]).

When narrowing the profession to the teacher, teachers’ job satisfaction has also been defined differently by researchers (e.g., [37,38,39,40]). Lortie considered teachers’ job satisfaction as an overall feeling of the work environment and work climate [37]. Similarly, Landy defined teacher job satisfaction as a teacher’s overall personal perception of the profession and the work environment in which he or she works [38]. Batten considered teachers’ job satisfaction as an implicit criterion that varies from person to person and from time to time [39]. Recent empirical research emphasizes the importance of both job stress and work environment as key contributors to teachers’ job satisfaction and their overall well-being [40]. It is an implicit measure of emotional attachment, identification, and willingness to stay in the school where they work. Recent scholars suggested that teacher job satisfaction is a feeling related to the intrinsic teaching role, including student achievements and teaching responsibilities [41].

Generally speaking, job satisfaction is closely related to an employee’s physical and mental health [42,43]. One study conducted a systematic review and meta-analysis of 485 studies with a combined sample size of 267,995 individuals, and the results revealed that job satisfaction is strongly associated with mental/psychological disorders [44]. Specifically, the strongest relationships were found between job satisfaction with burnout, self-esteem, depression, and anxiety [45,46,47]. A modest correlation between job satisfaction and subjective physical illness was also found [48,49,50]. By conducting a meta-analysis of work–family conflict research over the past 20 years, researchers found that job satisfaction is also significantly correlated with an employee’s satisfaction with their family livelihood, thus job satisfaction is a strong predictor of overall life satisfaction and holistic well-being [51].

For teachers, researchers found that emotional labor strategies such as surface acting can increase burnout and lower job satisfaction, while deep acting or expressing naturally felt emotions tends to improve job satisfaction and reduce burnout [52]. Job satisfaction is also significantly related to their teaching performance, including their teaching involvement, commitment, and motivation. In addition, teachers’ job satisfaction can be analytically significant to students and schools [53]. In connection with the research goal of this dissertation, it is noteworthy noting that teachers’ job satisfaction is highly positively correlated with teachers’ retention rate [54,55,56]. Other researchers emphasize the significant impact of psychological coping strategies on job satisfaction among teachers [57]. Their research highlights that adaptive copers, who effectively utilize problem-solving and seek social support, experience higher job satisfaction. In contrast, teachers with less adaptive coping profiles, such as problem-avoidant or social-withdrawal copers, report lower job satisfaction, which is associated with increased emotional strain, psychological distress, and quitting intentions [58]. These findings underscore the importance of fostering effective coping mechanisms to enhance job satisfaction, which is crucial not only for teacher retention but also for maintaining high educational standards and teacher mental well-being.

### 2.2. Teachers’ Self-Efficacy

American psychologist Albert Bandura noticed that there is a mechanism that plays a huge role in people’s lives, a mechanism whereby people’s beliefs have the ability to influence the events in their lives [59]. This suggests that individuals’ beliefs regarding their ability to control their behavior and incidents are particularly vital, and this kind of belief is referred to as self-efficacy [60]. Self-efficacy is a crucial component of the social cognitive theory created by Bandura [60]. The social cognitive theory consists of two parts, including learning theory and motivation theory. Learning theory focuses on the formation process of human behavior, while motivation theory focuses on the control and regulation of human behavior. According to Bandura, self-efficacy is the core concept of motivation theory as it is a subjective judgment and feeling of an individual’s ability [60]. Importantly, in the context of occupational psychology, self-efficacy is now viewed as a foundational mechanism for promoting emotional resilience, adaptive functioning, and mental health stability in high-stress professions such as teaching. While self-efficacy has traditionally been examined as a predictor of instructional quality or career commitment, recent studies in occupational health psychology highlight its role in mitigating symptoms of anxiety, emotional exhaustion, and work-related psychological disorders [22,26]. Particularly in emotionally demanding professions like teaching, high self-efficacy can reduce the physiological stress response, strengthen adaptive coping, and foster psychological recovery from daily work strain—components central to a healthy working life. Therefore, a more health-centric conceptualization of self-efficacy provides a valuable lens for understanding teacher well-being and retention.

Researchers after Bandura have developed various understandings of self-efficacy (e.g., [61,62,63]). Specifically, one study stated that self-efficacy is an employee’s recognition of or confidence in his or her ability to perform a task in a particular circumstance [62]. It helps an employee mobilize resources sufficiently and to make one’s motivation executed. Self-efficacy is a certain ability that an individual possesses when faced with a universal task [63]. Another study considers self-efficacy to be the corresponding mental reflection of a person in a given situation [61]. From the above-mentioned definitions of self-efficacy, we can see that self-efficacy is closely related to the self-assessment of one’s competency. In a nutshell, self-efficacy is the recognition and confidence that an employee has in his or her own performance in a given context; people engaged in different jobs may convey different meanings to self-efficacy. Beyond the workplace, self-efficacy has also been identified as a key determinant of behavioral health engagement [64].

The discussion above provided a broad and occupation-neutral lens on the concept of self-efficacy. Narrowing the perspective to the profession of teaching, teachers’ self-efficacy is defined differently by researchers (e.g., [65,66]) The foremost example is built upon the concept made by Bandura: teachers’ self-efficacy is a teacher’s beliefs in his or her ability to perform specific teaching tasks in a given situation [27]. Some researchers have also linked teachers’ self-efficacy to students’ achievements. For instance, teachers’ self-efficacy is a teacher’s belief that he or she can influence and help students. In addition, teachers with high self-efficacy believe they have a positive impact on student learning [66]. One early study suggested that teachers’ self-efficacy is a sense of satisfaction and affirmation of their teaching effectiveness; it is a feeling that they realize that their hard work pays off when they see their students succeed [65].

In the field of education, teachers’ self-efficacy is a crucial factor that many researchers found to be closely related to other outcomes including teachers’ teaching effectiveness, professional dedication, collective efficacy, burnout, teaching performance, students’ motivation and accomplishments, obstacles in teaching, stress level, and job satisfaction, etc. For instance, one study conducted a meta-analysis that demonstrated a significant relationship between teachers’ self-efficacy and their teaching effectiveness, particularly in evaluated teaching performance, indicating that teachers with higher self-efficacy tend to be rated as more effective by their supervisors and peers [67]. Another study conducted a meta-analysis and confirmed that self-efficacy is a moderate predictor of teacher commitment, highlighting its pivotal role in reducing teacher attrition and increasing long-term professional dedication [68]. A further quantitative study of 244 primary and secondary school teachers’ responses to a questionnaire and found that teachers’ self-efficacy is a multidimensional structure that is closely related to teachers’ collective efficacy and teacher burnout [69]. This reinforces its dual importance as both a pedagogical and occupational mental health variable.

For students’ learning, teachers’ self-efficacy is significantly related to their teaching performances as well as their students’ motivation and accomplishments [70,71]. By studying a sample of 247 Spanish secondary teachers, one study revealed that teachers with low self-efficacy are likely to encounter greater obstacles in teaching and higher levels of stress, and vice versa [72]. These cognitive and emotional stressors may contribute to long-term psychological fatigue, a known risk factor for teacher burnout. Last but not least, teachers with high self-efficacy tend to perceive job satisfaction (e.g., [17]).

Highlighting the importance of conceptualization in teacher self-efficacy, one study demonstrates that the impact of teacher self-efficacy on student outcomes varies significantly depending on its measurement [73]. Their findings suggest that while student-specific teacher self-efficacy positively correlates with academic achievement, general teacher self-efficacy may show negative associations at the classroom level, underscoring the critical role of teacher self-efficacy in educational settings. Building on these insights, another study extends the discourse on teacher self-efficacy by examining its interaction with school context over the trajectory of a teacher’s career [74]. Their longitudinal analysis reveals that both the school environment and perceived work demands significantly moderate the influence of teacher self-efficacy on teacher–student relationships. This study highlights that supportive school contexts can enhance the positive effects of teacher self-efficacy, particularly in fostering enduring and effective teacher–student interactions. Conversely, excessive work demands can diminish these benefits, underscoring the necessity of considering both individual and contextual factors in the application of teacher self-efficacy theories to educational practice. Further exploring the implications of teacher self-efficacy in the dynamic landscape of the teacher’s side; one study enhances our understanding of teacher self-efficacy by examining its relationship with work engagement, and their research reveals that stable levels of teacher self-efficacy robustly predict work engagement, affirming teacher self-efficacy’s foundational role in maintaining teacher motivation and effectiveness [75]. Given its strong association with emotional engagement, perceived agency, and instructional self-confidence, teacher self-efficacy may also serve as a protective psychological factor within teacher health promotion frameworks. These studies collectively emphasize that while teacher self-efficacy’s influence is profound, it is also shaped by its conceptualization and the contexts within which it operates, highlighting the necessity of nurturing teacher self-efficacy to achieve optimal educational outcomes. Recent evidence [76,77,78] highlights how post-COVID health impairments—such as fatigue, sleep disturbances, and legal–institutional challenges—may contribute to burnout and reduced job satisfaction among educators.

### 2.3. Framework of the Current Meta-Analysis

From the previous sections, it is clear that both teachers’ self-efficacy and job satisfaction are critical constructs that can affect the teaching profession in many ways. They were often validated to have a significant positive relationship with each other from studies across the world (e.g., [79,80,81]). One study found significant positive relationships between teachers’ self-efficacy and job satisfaction by conducting a structural equation modeling analysis to investigate 781 Western Australian high school teachers from 29 schools [80]. Another study conducted a correlation test with 83 teachers (46 primary school teachers, 27 middle school teachers, and 10 secondary school teachers) from different branches in Turkey, and a significant positive relationship was found between teachers’ self-efficacy and job satisfaction [79]. A third study’s research on 105 Louisiana agriculture teachers revealed a substantial and positive relationship between teachers’ self-efficacy and job satisfaction [81].

Although researchers from different continents of the world have found a positive and significant correlation between local teachers’ self-efficacy and job satisfaction, in the literature searching process, something to the contrary was also found [82,83]. Specifically, one study concluded in a study of 121 Irish teachers from eight different elementary schools that there was an insignificant negative correlation between teachers’ self-efficacy and their job satisfaction (r = −0.04, *p* > 0.05) [82]. Similarly, another study assessed 208 U.S. middle school teachers’ self-efficacy and their job satisfaction, and a non-significant and negative correlation between the two was found (r = −0.01, *p* > 0.05) [83].

According to social cognitive theory, self-efficacy is context-dependent. Teachers at different school levels face distinct challenges—elementary teachers often manage behavior and emotional development, while high school teachers deal with academic pressure and subject expertise. These contextual differences influence how self-efficacy is formed and how it affects job satisfaction [14]. The school level at which teachers work, such as elementary, middle, and high school, often experience varying levels of self-efficacy and job satisfaction, as well as their correlation. This claim is aligned with the work that highlighted the need to consider specific school contexts when assessing the role of self-efficacy in job satisfaction [84]. These variations can be attributed to the unique challenges and demands associated with each educational setting. For instance, elementary school teachers may have higher self-efficacy in classroom management and student engagement due to the nature of their interactions with younger children [85]. In contrast, high school teachers might face different stressors related to adolescent behavior and academic pressures, which can impact their job satisfaction and perceived self-efficacy differently, as suggested by a previous study [14]. Additionally, the impact of school climate plays a critical role in teachers’ perceived self-efficacy and their job satisfaction [80].

Different subjects place varied cognitive and emotional demands on teachers. For example, STEM subjects often involve high-stakes testing and abstract content, which may challenge self-efficacy, while subjects like literature and the arts rely heavily on student interaction, enhancing teachers’ sense of influence and control. This aligns with the task-specific nature of self-efficacy and its impact on satisfaction [86], as teachers who feel less confident in their abilities to teach these subjects may experience lower job satisfaction [14]. Subjects that rely heavily on student engagement, such as literature or the arts, may lead to higher self-efficacy for teachers who can manage classroom dynamics effectively and engage students. Teachers in these fields may feel more satisfied with their jobs when they perceive they are fostering creativity and discussion. Conversely, subjects that demand more rote memorization or factual teaching, like history or science, may affect self-efficacy differently due to less dynamic interactions (Moè et al., 2010 [86]). Subjects that demand extensive grading or preparation, such as language arts, where teachers must evaluate essays and written assignments, may lead to increased stress and workload, impacting both self-efficacy and job satisfaction. High workloads may decrease job satisfaction if teachers feel overwhelmed by the volume of work despite their self-efficacy in instructional strategies [14].

Language environment shapes both instructional demands and cultural expression of self-efficacy. Teachers in English-speaking contexts may face more standardized assessments and accountability pressure, which can constrain perceived autonomy and reduce self-efficacy, impacting satisfaction [87]. In studies exploring the relationship between teachers’ self-efficacy and job satisfaction across different national contexts, cultural and linguistic factors have been shown to play a significant role [88,89]. For instance, research involving data from 23 countries found a positive correlation between self-efficacy and job satisfaction, with variations in cultural value orientations, such as collectivism versus individualism, influencing how self-efficacy perceptions were expressed [90]. Similarly, a study on teachers in English-medium schools in the Dominican Republic showed that those in English-speaking contexts reported lower self-efficacy and job satisfaction compared to their counterparts in Spanish-medium schools [87].

Teachers in developed countries generally benefit from better infrastructure, training, and support, fostering stronger self-efficacy. In contrast, teachers in developing countries may struggle to build efficacy due to systemic challenges. Social cognitive theory suggests that enabling environments support efficacy formation, thereby enhancing job satisfaction [12]. A country’s economic level may directly influence teachers’ income, school infrastructure, and work environment, which in turn affects their self-efficacy and job satisfaction. Teachers in higher-income countries tend to have better access to educational resources, administrative support, and professional development opportunities. Due to income levels and various incentive measures, teachers perceive the teaching profession as providing a secure future. They are more inclined to recognize the possibility of career advancement and strive to achieve this goal. Additionally, the job security provided by the educational system motivates teachers to actively engage in their work, and a positive attitude towards the work environment helps improve job satisfaction [12]. Educational systems in the Southern Hemisphere often suffer from a mismatch between curricula and local needs, limited resources, and systemic instability. These contextual constraints can undermine teachers’ self-efficacy, leading to lower job satisfaction. This reflects Bandura’s emphasis on the role of environmental influences in efficacy development. For instance, in the Southern Hemisphere, educational systems often fail to adequately meet the real needs of learners. Many countries tend to adopt educational models from the Northern Hemisphere without making the necessary local adaptations, resulting in curricula that do not align with the actual skills and needs of students. This is especially evident in countries like South Africa and Madagascar, where the education systems are not sufficiently tailored to their socioeconomic realities. As a result, learners are not provided with the relevant opportunities to develop the competencies they need to function effectively according to their true educational needs. This lack of localization hampers the overall effectiveness of these educational systems [91]. This situation may lead to changes in school climate, which could affect the correlation between teachers’ self-efficacy and their job satisfaction [92].

Before listing the specific moderators, it is important to provide a theoretical rationale for why these variables may influence the relationship between teachers’ self-efficacy and job satisfaction. Grounded in social cognitive theory [16], the following factors are proposed to moderate the strength of this relationship due to their potential influence on how efficacy beliefs are formed, enacted, and supported in various teaching contexts (see Table 1).

Based on the theoretical considerations presented in Table 1, the current meta-analysis identified the following moderators to examine the possible moderation effect they may have on the results:

- Teacher-level characteristics (school level at which teachers work, subject that teachers teach).
- Country-level characteristics (Southern Hemisphere countries vs. Northern Hemisphere countries; English native-speaking countries vs. non-English native-speaking countries, developed vs. developing countries).

We conducted an extensive search of published research on the relationship between teachers’ job satisfaction and their self-efficacy to compare how self-efficacy influences job satisfaction across different teaching environments, as well as the moderation effects certain characteristics may introduce. We also examined the heterogeneity in the results of these studies to assess whether researchers have drawn different conclusions regarding the association between self-efficacy and job satisfaction. Collectively, this study addressed the following three research questions:

RQ 1: What is the overall estimated average strength of the relationship between teachers’ self-efficacy and their job satisfaction?

RQ 2: How heterogeneous (i.e., in effect sizes, in type of teacher studied, in type of studies, etc.) are these studies on the overall relationship between teachers’ self-efficacy beliefs and job satisfaction levels?

RQ 3: To what extent do teacher-level characteristics (school level at which teachers work, subject that teachers teach) and country-level characteristics (English native-speaking countries vs. non-English native-speaking countries, developed vs. developing countries, Southern Hemisphere countries vs. Northern Hemisphere countries) moderate the effect?

## 3. Methods

Meta-analysis refers to the quantitative synthesis of the results of multiple independent studies with a common or similar research purpose. In this way, the researchers can dissect the differences between extant studies and comprehensively evaluate the prior findings [93]. In addition to the fact that meta-analysis can be used to quantitatively synthesize the results of previous studies, it can also examine the differences between the findings in different research contexts, avoid bias in the results due to factors such as sample source and geographical differences in individual studies, and examine the issue from a more comprehensive perspective to draw more reliable conclusions [94]. For instance, researchers can combine a scoping review and meta-analysis to analyze the utility of meta-analytic methods in identifying overall effects and moderator impacts across educational contexts [95]. For this reason, and in conjunction with the purpose of this dissertation, this study aims to exhaustively search and analyze previous studies that explored the relationship between teachers’ job satisfaction and their self-efficacy.

### 3.1. Literature Searching and Screening

This study adopted the method of computerized database search to conduct the literature search. Databases being searched include ERIC (Education Resources Information Center), ProQuest, Web of Science, and Google Scholar. Publications that were collected include peer-reviewed journals and dissertations with the following terms in their titles or abstracts:

[(“self-efficacy” or “teachers’ self-efficacy”) AND (“job satisfaction” or “teachers’ job satisfaction”) OR (“relationship between self-efficacy and job satisfaction” or “relationship between teachers’ self-efficacy and teachers’ job satisfaction” or “relationship between teacher self-efficacy and job satisfaction” or “teacher self-efficacy and job satisfaction” or “self-efficacy and teachers’ job satisfaction”)].

In the first round of screening, the title and abstract of the article were read first, and articles that fit the topic of this meta-analysis were retained. After this screening step, the review articles, introductory articles that describe teachers’ self-efficacy and/or job satisfaction, and articles that include only one aspect of research (e.g., examining teacher job satisfaction or self-efficacy alone) were excluded. After this step, 2517 articles were identified.

Then, the 2517 articles were screened one by one according to the following criteria. After the literature screening, all eligible studies were retained and coded.

### 3.2. Inclusion and Exclusion Criteria

The inclusion and exclusion criteria to decide whether a study should be included are as follows:

(1) The studies should be related to self-efficacy and teachers’ job satisfaction.
(2) Participants of eligible studies should be primary school teachers, elementary school teachers, middle school teachers, high school teachers, college teachers, etc.
(3) The studies should use empirical approaches to examine the relationship between teachers’ self-efficacy and their job satisfaction.
(4) The studies should report sufficient data (N, r, SE, etc.), and the quantitative data reported in the study are enough for calculating the effect size.
(5) Studies exhibiting outlier SD or effect sizes were excluded from the analysis. This exclusion was necessitated by the possibility that the observed within-study variances in these cases might be attributable to sampling errors rather than reflecting genuine variability. The incorporation of such studies into the meta-analysis could compromise the integrity and validity of the resultant findings.
(6) Studies could have been conducted in any region, but the data should have been written in English.

**Figure 1:**
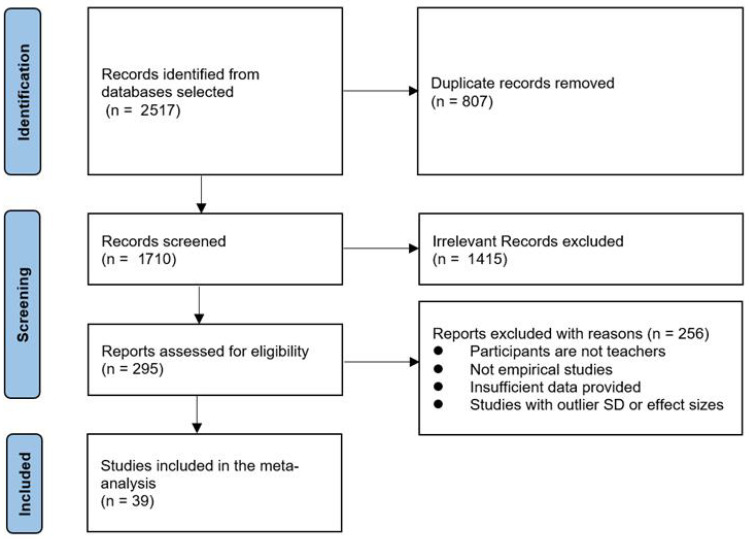
PRISMA flow diagram of the study selection.

After the initial screening, we identified 2517 articles from the selected databases. In the first round of duplicate identification, we removed 807 duplicate records. Subsequently, we screened 1710 records and excluded 1415 relevant records based on the content. Then, we assessed 295 articles for eligibility and ultimately excluded 256 reports for various reasons, including participants not being teachers, some being non-empirical studies, insufficient data provided to calculate effect sizes, and studies with outlier standard deviations or effect sizes. After screening out all articles that did not meet the inclusion criteria, we ended up with 39 eligible studies, and the inclusion process based on the PRISMA flow diagram [96] is shown in Figure 1.

### 3.3. The Characteristics of Selected Studies and Data Coding

The current meta-analysis includes items in the coding sheet to describe the important study features according to the research setting and the information provided by all selected studies. The characteristics that are coded and their classification criteria are based on the following:

(1) Country: The country in which the research was conducted/participating teachers’ country of residence.
(2) Year of publication: The year of publication of the article.
(3) School level at which teachers worked: The school level the teacher teaches was identified based on the information provided in the article. According to the level of the schools to which the teachers belong, this study categorizes the selected studies into pre-high school (including primary, elementary, and middle schools) and post-high school (including high schools and colleges, or cases where teachers taught at multiple levels including high school/colleges).
(4) Subject taught: The main subject taught by the participants. This study divides the selected studies into two categories: those involving teachers of all subjects and those focusing on teachers of a specific subject (e.g., English, Agriculture, etc.).
(5) Hemisphere: The location of the research was categorized based on whether it was conducted in the Southern or Northern Hemisphere. This study groups the research locations into two categories: Southern Hemisphere and Northern Hemisphere, depending on the geographic location of the country where the study took place.
(6) Language: This refers to whether the research was conducted in an English-speaking country or if the participating teachers’ primary language was English. The study categorizes the selected research into two groups: those conducted in English-speaking countries and those conducted in non-English-speaking countries.
(7) Development Status: The classification of whether the research was conducted in a developed country or if the participating teachers resided in a developed country was based on Organization for Economic Co-operation and Development (OECD) membership. This study categorizes the countries where the research was conducted into developed countries (OECD member countries) and developing countries (non-OECD member countries).

### 3.4. Effect Size Evaluation

The data analysis was performed using statistical software R 4.4.1. We converted the correlation coefficient to Fisher’s Z to calculate the effect size [97], along with its 95% confidence interval (CI), where *p* < 0.05 was determined as the level of significance. Because the correlation coefficients of these 39 studies were obtained from independent and different datasets, a random-effects model was primarily selected for meta-analysis, pending the heterogeneity test.

A forest plot was employed to show the effect size of each independent sample.

### 3.5. Effect Size Homogeneity

To test the degree of homogeneity of the selected samples, Q statistic [98] and I^2^ [99] were computed, and their results were explained.

### 3.6. Publication Bias and Sensitivity Test

Publication bias implies that the sample for meta-analysis may not be fully representative, especially when studies with non-significant results are underreported. To assess the robustness and fairness of the results, we conducted both trim-and-fill analysis [100] and Egger’s regression test [101]. These procedures not only help evaluate the risk of publication bias but also serve as a form of sensitivity analysis, allowing us to test whether the inclusion of potentially missing studies would substantially alter the overall effect size. This approach strengthens the credibility and generalizability of the meta-analytic findings.

## 4. Results

### 4.1. Overview of the Selected Studies

Of the 39 studies selected, we can see the globality of this topic as teachers from all five continents participated in the research on this topic. In terms of the country in which the participants were selected, the United States holds the top spot with eight studies (20.5%). Turkish and Italian teachers also participated in a substantial number of studies, with six (15.4%) and five (12.8%) studies, respectively. Altogether, the participants from the top three countries contributed 19 studies (48.7%), and 16 other countries comprise the remaining half.

When referring to the school levels the teacher participants were teaching with when the study was conducted, teachers in grades K-12 are the main force who contributed to 35 studies (89.7%). Three studies did not specify the school level of the participating teachers, and three studies had college teachers involved (two of which blended college and high school teachers).

The subject taught by teachers is not the focus of studies examining the relationship between teachers’ self-efficacy and their job satisfaction. Of the 39 studies selected, only 4 studies (10.3%) indicated the specific subject that the participating teachers taught. As for the research topic of the current dissertation, although the author was able to identify two studies that explored the possible relationship between STEM teachers’ self-efficacy and job satisfaction [56,102], neither study was included in this meta-analysis due to lack of required statistics. Specifically, one study investigated the differences in job satisfaction and its influencing factors between STEM and non-STEM novice teachers via the School and Staffing Survey (SASS) 2011–2012 dataset [56]. The statistical methodologies they employed include multiple regression analysis and Z-tests. Similarly, another study examined the effects of school climate and self-efficacy on mostly STEM teachers’ job satisfaction by employing structural multigroup invariance analysis of the Teaching and Learning International Survey (TALIS) 2018 dataset [102]. However, neither study provided a correlation matrix nor Pearson’s correlation specifying the correlation between job satisfaction and self-efficacy. Of the four studies that specified the subjects that teacher participants taught, two were English, one was Agriculture, and one was Physical Education.

The characteristics (study number, author and publication year, correlation coefficient, sample size, country of participants, subject participating teachers taught, and publication type of the article) of each study are detailed in Table 2.

### 4.2. Overall Effect Sizes

**Figure 2:**
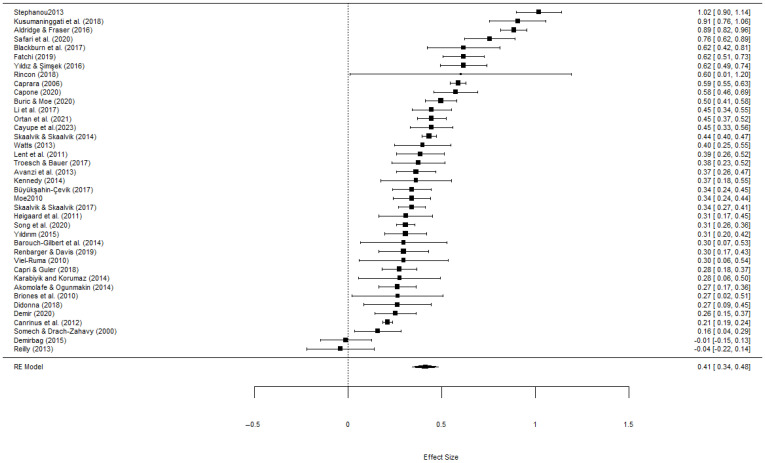
Forest plot of 39 studies’ effect sizes [12,79,80,81,82,83,84,86,87,103,104,105,106,107,108,109,110,111,112,113,114,115,116,117,118,119,120,121,122,123,124,125,126,127,128,129,130,131,132].

The 39 effect sizes (Fisher’s Z) identified in this meta-analysis ranged from −0.04 to 1.02, of which 37 (94.9%) were found to be positive, which provides partial evidence for the positive correlation between teachers’ self-efficacy and their job satisfaction. As shown in Table 3, the estimated average effect size under the overall random effect model is 0.41 (*p* < 0.001) and 95% CI is [0.34, 0.49], which indicated a small to medium effect [133]. This means the estimated average effect size was statistically significant, and the null hypothesis that teachers’ self-efficacy and their job satisfaction are not correlated is rejected. The forest plot is shown in Figure 2.

### 4.3. Effect Sizes’ Homogeneity

The homogeneity test shows that the null hypothesis of effect size homogeneity is violated (Q (38) = 802.07, *p* < 0.001, I^2^ = 97%). These results indicate that the variation in the effect sizes is high among these studies [134]. The high heterogeneity of the results from extant studies examining the relationship between teachers’ self-efficacy beliefs and job satisfaction warrant further investigation to find out the specific situation of this relationship in different contexts, such as special education teachers, teacher of gifted children, STEM teachers, etc.

### 4.4. Results of the Publication Bias and Sensitivity Analyses

**Figure 3:**
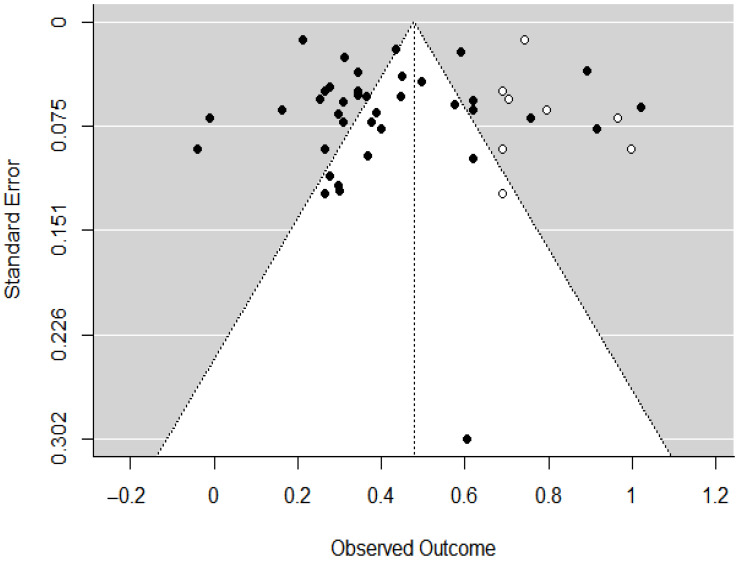
Funnel plot of trim-and-fill analysis. Note: each black dot in this figure represents an effect size of the included study, and each white dot represents a computer-generated study for symmetry [135].

Only eight imputed values were identified in the funnel plot through the trim-and-fill analysis (see Figure 3). The majority of data points were symmetrically distributed around the mean, indicating a relatively low risk of publication bias. Furthermore, Egger’s regression test produced a result of t(37) = 1.13, *p* = 0.27, which is not statistically significant, suggesting no evidence of serious publication bias [101]. Importantly, the overall effect size remained statistically significant and comparable after accounting for these imputed values. As such, these procedures also function as a sensitivity test, confirming that the key meta-analytic conclusion remains robust even when accounting for potentially missing or biased studies.

### 4.5. Moderator Analysis

To determine the extent to which the relationship between self-efficacy and teachers’ job satisfaction is moderated by regional and institutional characteristics, a series of stratified analyses were performed.

### 4.6. School Level at Which Teachers Worked

Pre-high school and after-high school account for 61.54% (k = 24) and 38.46% (k = 15) of the studies, respectively. As shown in Table 4, the effect size for after-high school (ES = 0.52) was larger than pre-high school (ES = 0.34), and the difference was significant (Q_between_ = 7.09, *p* < 0.01).

### 4.7. Subject Taught

Studies conducted for all subjects and single subject account for 89.74% (k = 35) and 10.26% (k = 4), respectively. The effect size for studies conducted for single subject (ES = 0.57) was larger than studies conducted for all subjects (ES = 0.39), but the difference was not significant (Q_between_ = 2.30, *p* > 0.05).

### 4.8. Hemisphere

Approximately 89.74% (k = 35) of the studies were conducted in the Northern Hemisphere countries, while 10.26% (k = 4) of the studies were conducted in the Southern Hemisphere countries. The effect size for studies conducted in the Southern Hemisphere countries (ES = 0.75) was larger than studies conducted in the Northern Hemisphere countries (ES = 0.38), and the difference was significant (Q_between_ = 9.03, *p* < 0.01).

### 4.9. Language

Approximately 76.92% (k = 30) of the studies were conducted in non-English-speaking countries, while 23.08% (k = 9) of the studies were conducted in English-speaking countries. The effect size for non-English-speaking countries (ES = 0.41) was approximately equal to non-English-speaking countries (ES = 0.41), but the difference was not significant (Q_between_ = 0.01, *p* > 0.05).

### 4.10. Development Status

Approximately 58.97% (k = 23) of the studies were conducted in developed countries, while 41.03% (k = 16) of the studies were conducted in non-developed countries. The effect size for non-developed countries (ES = 0.44) was higher than for developed countries (ES = 0.39), but the difference was not significant (Q_between_ = 0.44, *p* > 0.05).

## 5. Discussion

Although we can see the globality of this topic—as teachers from five continents participated in the research focusing on the correlation between self-efficacy and job satisfaction since 2000—the country homogeneity and domination can still be seen in the participating countries. Despite the fact that these 39 studies examined teachers from 18 countries, half of the studies focused on teachers from 3 countries: the United States, Turkey, and Italy. This imbalance may limit global health equity efforts to understand and support the psychological well-being of teachers worldwide. The United Nations reported that at least 114 countries and territories around the world face a severe shortage in the teaching workforce [136]. Given the growing concern over the mental health crisis in educational professions globally, we call for researchers from all over the world to conduct studies focusing on teachers’ self-efficacy and job satisfaction, in order to identify causes of teacher attrition and propose psychologically sustainable interventions.

When referring to the school levels the teacher participants were teaching when the study was conducted, some findings are thought-provoking. For example, only half (n = 19) of the studies limited the grade levels of participating teachers to a specific school level (i.e., elementary school, middle school, high school, or college). Sixteen studies combined teachers from at least two school levels. As previous studies found significant differences in teachers’ perceptions of job satisfaction across grade levels, distinguishing between school levels is particularly critical [15,137]. Teachers’ exposure to stressors and support systems can vary greatly by school level, affecting their occupational mental health. Thus, we urge future researchers to make school level a key consideration when investigating this topic.

The subject taught by participating teachers is not a main focus in most studies on the self-efficacy–job satisfaction relationship. Of the 39 studies selected, only 4 (10.5%) specified the subject: 1 on Physical Education, 1 on Agriculture, and 2 on English. Although two studies specifically targeted STEM teachers, neither met the inclusion criteria due to missing statistics. This omission is concerning, given evidence that STEM teachers report higher emotional exhaustion and lower retention rates, suggesting greater need for psychosocial support [138].

### 5.1. Significant Positive Correlation

Of the 39 effect sizes (Fisher’s Z), 37 (94.9%) were positive, and the overall estimated average effect size was 0.41 (*p* < 0.001), 95% CI [0.34, 0.49]. These results support a statistically significant positive relationship between teachers’ self-efficacy and job satisfaction, reinforcing the social cognitive theory [16], which posits that higher self-efficacy increases motivation and persistence, thereby enhancing job satisfaction. This also supports the argument that teacher self-efficacy is not just a cognitive construct but a protective psychological resource that promotes workplace well-being.

### 5.2. Heterogeneity of Effect Sizes

Despite an overall positive relationship, the high heterogeneity observed in effect sizes (I^2^ = 97%) indicates variability in how self-efficacy impacts job satisfaction across different contexts. This variability could stem from differences in cultural, educational, and institutional settings, as highlighted by the divergent results in studies conducted in various countries and school levels (e.g., [84,88]). This means that when interpreting and generalizing research findings, it is essential to account for these contextual factors. For instance, differences in national education policies, sociocultural values, resource allocation, and societal recognition of the teaching profession can all influence the relationship between self-efficacy and job satisfaction. Therefore, future research should further explore how these contextual factors moderate this relationship to provide more universally applicable and adaptable conclusions. In the meantime, this heterogeneity highlights the need for context-specific strategies when designing interventions to enhance teachers’ self-efficacy and job satisfaction. A one-size-fits-all approach may not be effective across all educational environments. Research should focus more on the specific challenges faced by teachers in different school levels (e.g., primary, secondary) and educational settings (e.g., rural vs. urban schools) to design targeted strategies for boosting self-efficacy, which can more effectively improve job satisfaction. In addition, the availability of occupational health support and stress management resources within educational institutions may either amplify or buffer this relationship.

### 5.3. Moderators

The results of the meta-analysis reveal that various factors may moderate the relationship between teachers’ self-efficacy and job satisfaction. Understanding these moderators is crucial for tailoring interventions that aim to enhance teachers’ well-being and improve educational outcomes. In this section, we discuss both significant and non-significant moderators identified in the study, along with possible reasons for these findings.

### 5.4. School Level at Which Teachers Work

The analysis shows that school level is a significant moderator, with different relationships observed between self-efficacy and job satisfaction depending on whether teachers work at pre-high school (e.g., elementary, middle school) or post-high school (e.g., high school, college) levels. Specifically, the effect size for after-high school teachers (ES = 0.52) was significantly larger than for pre-high school teachers (ES = 0.34), suggesting that self-efficacy has a more substantial impact on job satisfaction among high school and college educators.

One potential reason for this finding is the differing nature of responsibilities and challenges faced by teachers at various educational levels. High school teachers often deal with more complex subject matter, higher academic standards, and the pressure to prepare students for college entrance exams and future careers. These responsibilities may increase stress levels but also provide opportunities for teachers to feel a strong sense of accomplishment and competence, enhancing job satisfaction when they feel effective in their roles [14,85]. In contrast, elementary school teachers may experience fewer subject-specific pressures and more routine classroom management tasks, which might result in a less pronounced correlation between self-efficacy and job satisfaction. This aligns with occupational health models suggesting that perceived control and task significance influence professional fulfillment.

### 5.5. Subject Taught

The analysis also explored whether the subject taught by teachers moderates the relationship between self-efficacy and job satisfaction. While studies focusing on single subjects (e.g., Physical Education, Agriculture, English) reported a higher effect size (ES = 0.57) compared to studies involving teachers of all subjects (ES = 0.39), the difference was not statistically significant.

The non-significant difference may be due to the diverse nature of teaching subjects. Teachers’ self-efficacy across various subjects could still hinge on similar core competencies, such as effective communication, classroom management, and student engagement, which are applicable to all teaching disciplines. Moreover, the relatively low number of studies focusing exclusively on single-subject teachers may limit the power to detect significant differences. It is also possible that subject-specific self-efficacy is moderated by other factors, such as the availability of subject-specific resources, professional development, and peer support, which were not controlled for in the current analysis.

### 5.6. Hemisphere

The effect size for studies conducted in Southern Hemisphere countries was significantly larger than that for Northern Hemisphere countries. Educational challenges and structural inequalities in school health systems may contribute to this disparity. In the Southern Hemisphere—particularly in developing countries—teachers often face resource limitations, larger class sizes, and higher student-to-teacher ratios. These conditions may lead teachers to rely more heavily on their self-efficacy to navigate daily stressors, thereby reinforcing the relationship between self-efficacy and job satisfaction. In resource-constrained environments, self-efficacy may act as a resilience mechanism, enabling teachers to feel fulfilled and competent despite systemic barriers [139].

Cultural attitudes towards education may also help explain this result. In many Southern Hemisphere countries, teaching is regarded as a respected and community-centered profession. This cultural valuation can magnify the impact of self-efficacy on job satisfaction, as teachers in these regions may feel a stronger sense of purpose and psychosocial reward. Research shows that when teachers are culturally supported, those with higher self-efficacy experience stronger job satisfaction because they perceive their contributions as meaningful and appreciated [110].

In addition, teachers in Southern Hemisphere countries often face additional stressors such as political instability, lower salaries, and insufficient governmental support. In such contexts, self-efficacy becomes even more crucial in maintaining job satisfaction. Studies have indicated that teachers with strong self-efficacy are better equipped to handle job-related stress and workload issues, which are common in under-resourced educational systems. This heightened reliance on self-efficacy to cope with adversity may strengthen the relationship between self-efficacy and job satisfaction [84].

### 5.7. Other Potential Moderators and Future Research Directions

While the current meta-analysis identified significant moderation by school level, other potential moderators—such as years of teaching experience, school climate, administrative support, and the socioeconomic status of the student population—could further illuminate the complex relationship between self-efficacy and job satisfaction. For instance, school climate has been shown to influence both self-efficacy and job satisfaction, with supportive, collegial, and psychologically safe environments improving teachers’ perceptions of control and success [80].

Poor school climate and lack of autonomy have been identified as risk factors for teacher burnout, suggesting the importance of embedding mental health-supportive frameworks into the organizational culture of schools. Future studies should integrate these variables to capture a more holistic picture of the conditions under which self-efficacy most strongly impacts job satisfaction.

### 5.8. Country and Cultural Context

The analysis indicates that the country of origin, particularly whether it is an English-speaking country, did not significantly moderate the relationship between self-efficacy and job satisfaction. Both English-speaking and non-English-speaking countries showed similar effect sizes (ES = 0.41), suggesting that the influence of self-efficacy on job satisfaction might be relatively consistent across different linguistic and cultural settings. This might be attributed to the universal aspects of teaching that transcend cultural boundaries. Core teaching competencies, such as classroom management, instructional delivery, and student engagement, are likely to be important regardless of cultural or linguistic differences. Additionally, global trends in education reform, such as a focus on teacher quality and student outcomes, might have standardized teaching expectations and practices across different countries, thereby minimizing cultural variations. Furthermore, many of the studies included in this meta-analysis were conducted in countries with established education systems, which may share similar standards and expectations for teachers, further reducing the potential for cultural divergence in the relationship between self-efficacy and job satisfaction.

### 5.9. Developed vs. Developing Countries

Interestingly, the study found that the effect size was slightly higher in non-developed countries (ES = 0.44) compared to developed countries (ES = 0.39), although this difference was not statistically significant. This marginal difference may reflect varying levels of structural support. In non-developed countries, where educational infrastructure and teacher training may be limited, self-efficacy becomes a critical internal resource for coping with uncertainty, stress, and task overload. Teachers with strong self-efficacy in these settings may experience higher job satisfaction despite resource limitations, using their belief in competence to maintain emotional stability and commitment.

In contrast, teachers in developed systems may have access to formal support structures such as mentoring, health counseling, and workload protection mechanisms, which buffer the effects of low efficacy and result in more moderate effect sizes. These findings suggest that the protective role of self-efficacy is more salient in psychologically demanding or under-supported environments.

Furthermore, teaching conditions across countries are shaped not only by economic development but also by the degree of policy localization and systemic alignment. In many developing countries—particularly in the Southern Hemisphere—there is often a mismatch between imported curricula and local socioeconomic contexts. Educational models transplanted from Northern systems without sufficient adaptation may fail to address the actual competencies needed by learners, weakening teachers’ perceived instructional relevance and reducing their self-efficacy and job satisfaction [91]. For instance, in countries such as South Africa and Madagascar, the misalignment between curriculum content and community needs has contributed to systemic instability, poor student outcomes, and teacher demoralization [91]. Yet, despite these structural challenges, the role of self-efficacy may become even more pronounced in such under-resourced contexts. As our analysis shows, teachers in developing countries often rely more heavily on internal psychological resources like self-efficacy to cope with stress, uncertainty, and workload demands. In contrast, teachers in high-income countries benefit from institutional supports—such as formal mentoring, job security, and career pathways—that buffer the impact of low efficacy and contribute more moderately to job satisfaction [12]. These cross-national differences highlight that while contextual constraints can erode efficacy beliefs, they can also amplify the importance of self-efficacy as a protective factor—underscoring the need to interpret the efficacy–satisfaction relationship through the combined lenses of educational governance, policy adaptability, and sociocultural expectations.

### 5.10. Integrating Self-Efficacy into the Occupational Health Framework

Despite its origins in social cognitive theory, self-efficacy has grown into a key construct within occupational health psychology, where it is valued for its buffering effects against job-related stress and mental health deterioration. Teaching, often marked by emotional labor and role overload, benefits substantially from protective psychological factors like self-efficacy, which can mitigate the onset of chronic stress conditions such as burnout, anxiety, and depression [3]. Thus, future research and intervention design should align more closely with holistic occupational health models, where self-efficacy is positioned alongside systemic resources such as workload management, institutional support, and access to mental health services. This shift will allow for the development of comprehensive mental health promotion strategies that go beyond skill-based teacher training.

## 6. Theoretical and Practical Implications

### 6.1. Theoretical Implications

This meta-analysis offers robust theoretical contributions to the study of teacher self-efficacy and job satisfaction by providing empirical confirmation of their significant positive association across diverse educational and cultural contexts. Grounded in Bandura’s social cognitive theory [16], our findings reinforce the understanding of self-efficacy as a central psychological construct that drives motivation, resilience, and occupational well-being. The results demonstrate that teachers with stronger beliefs in their instructional abilities tend to report greater job satisfaction, underscoring self-efficacy as a crucial resource for sustaining teacher morale and effectiveness.

Moreover, the moderation analyses enrich theoretical discourse by highlighting the contextual nature of self-efficacy. The stronger correlation observed among post-high school educators suggests that greater autonomy and task complexity enhance the salience of self-efficacy in shaping satisfaction [14]. Similarly, the heightened effect sizes found in Southern Hemisphere studies (e.g., [91,139]) emphasize how structural challenges may amplify the importance of efficacy beliefs in coping with occupational stress. These findings support the conceptualization of self-efficacy—not as a fixed trait but as a dynamic, context-sensitive construct. However, a critical gap remains in understanding subject-specific self-efficacy: few studies have disaggregated effects by teaching domain despite varying instructional demands. Future theoretical models should more systematically explore how self-efficacy varies across disciplines and interacts with institutional supports and stressors.

Beyond educational outcomes, our findings underscore the value of integrating psychological constructs like self-efficacy into occupational health discourse. By positioning self-efficacy as a core protective factor within the broader mental health framework, this research supports efforts to design school-based mental health interventions that address both individual agency and systemic stressors. Such an approach is particularly important for preventing burnout, enhancing teacher retention, and improving the psychological resilience of educators in high-demand environments.

### 6.2. Practical Implications

Given the pressing global concerns regarding teacher attrition, mental health, and workforce sustainability, the findings from this meta-analysis provide timely and practical guidance for educational stakeholders. Educational policymakers should prioritize the inclusion of self-efficacy-building strategies in national teacher development frameworks, particularly in high-burnout sectors such as STEM and vocational education. School principals and administrators are encouraged to foster emotionally supportive school climates by promoting teacher autonomy, constructive feedback, and peer collaboration, all of which have been shown to strengthen self-efficacy and enhance job satisfaction [80]. Occupational mental health managers should incorporate self-efficacy assessment and personalized coaching into teacher wellness programs, especially in under-resourced settings where burnout risks are elevated.

Meanwhile, teacher education institutions should embed efficacy-enhancing practices—such as mastery experiences, vicarious learning, and guided feedback—into both pre-service and in-service training, in alignment with Bandura’s four sources of efficacy beliefs [16]. In low-income or high-pressure contexts, targeted investment in peer mentoring and localized professional learning communities can reinforce collective efficacy and psychological resilience. Taken together, these strategies underscore that fostering teacher self-efficacy is not merely an individual-level intervention, but a systemic priority essential for building a committed, competent, and mentally resilient education workforce.

## 7. Limitations and Future Directions

This study has several limitations that should be acknowledged. Firstly, the scope of this meta-analysis was limited to studies published in English and those conducted after the year 2000. This criterion might exclude relevant research published in other languages or before 2000, which could have provided a more comprehensive understanding of the topic. Future studies should consider including non-English literature and expanding the time frame to capture a broader range of data. Secondly, limited by the information provided in the selected studies, this analysis did not differentiate between various educational settings, such as urban versus rural schools, which may present different challenges and resources affecting teachers’ self-efficacy and job satisfaction. Further research should incorporate these variables to provide a more nuanced understanding of how different teaching environments impact the relationship between self-efficacy and job satisfaction. Additionally, the focus on general teacher populations means that specific groups, such as special education teachers, teachers of gifted children, STEM teachers, etc., were under-represented. Given the unique challenges faced by these educators, particularly regarding specific pressures and the high turnover rates in these fields, future research should specifically target the relationship between self-efficacy and job satisfaction among these teachers.

Moreover, this meta-analysis highlights the need for longitudinal studies that track changes in self-efficacy and job satisfaction over time. Such studies could offer deeper insights into how these variables interact throughout a teacher’s career, revealing critical periods when interventions might be most effective. Additionally, exploring other potential moderators, such as gender, years of teaching experience, and school climate, could provide a more comprehensive understanding of the complex interplay between self-efficacy and job satisfaction.

## 8. Conclusions

This meta-analysis has demonstrated a statistically significant positive correlation between teachers’ self-efficacy and job satisfaction across a global scale, highlighting the universal relevance of these two constructs in the teaching profession. The findings underscore the importance of self-efficacy as a key factor contributing to teachers’ overall job satisfaction, suggesting that initiatives aimed at enhancing teachers’ confidence in their professional abilities could serve as a viable strategy to improve job satisfaction and reduce turnover rates. This is especially relevant within the broader discourse of occupational well-being and mental health in education, where self-efficacy functions as a protective psychological resource against stress and burnout. These insights are particularly crucial in light of the ongoing global challenge of teacher shortages, which have been exacerbated by high attrition rates linked to job dissatisfaction and unmanageable work demands.

Given the significant positive correlation found in this study, it becomes evident that empowering teachers by fostering a strong sense of self-efficacy could lead to more committed, motivated, and satisfied educators. Enhancing self-efficacy may not only boost perceived professional competence but also strengthen psychological resilience in high-pressure teaching environments. This, in turn, may improve teaching quality, elevate student outcomes, and reinforce the health and sustainability of educational systems. These findings advocate targeted interventions—such as professional development programs, mentorship opportunities, and emotionally supportive school climates—that bolster teachers’ self-efficacy and, consequently, their job satisfaction. From a systems health perspective, such interventions may reduce psychological distress, prevent burnout, and promote long-term retention in the teaching workforce.

Meanwhile, this meta-analysis also revealed considerable heterogeneity in the results, suggesting that the strength of the relationship between self-efficacy and job satisfaction varies depending on contextual factors. Notably, differences in effect sizes were observed based on the school level at which teachers operate, with stronger correlations reported among high school and college educators compared to those in elementary and middle school settings. This implies that emotional labor, role complexity, and cognitive workload differ by educational stage, influencing the degree to which self-efficacy enhances satisfaction. Such variability underscores the need for differentiated strategies that address school-level challenges and tailor self-efficacy interventions accordingly.

These findings call for more nuanced, international, and context-specific research to fully understand the factors that influence the correlation between teachers’ self-efficacy and job satisfaction. Future studies should examine this relationship across diverse cultural settings, educational levels, and subject areas. Incorporating psychosocial dimensions, such as teacher stress profiles, emotional regulation, and workplace support systems, may enrich the understanding of these dynamics. This approach can help identify the unique challenges and protective resources available to different teacher subgroups, offering guidance for the design of interventions that are both effective and context-sensitive.

In conclusion, the evidence presented in this meta-analysis affirms the significant correlation between teachers’ self-efficacy and job satisfaction, reinforcing the importance of cultivating a positive sense of efficacy among educators. Doing so not only enhances job satisfaction and retention but also contributes to the emotional well-being and mental health of teachers—a cornerstone of any resilient educational system. This reinforces the critical role that teacher development and support play in the broader educational ecosystem, emphasizing the need for policies and practices that prioritize teacher empowerment, mental health, and long-term engagement. As we move forward, continued cross-disciplinary research and evidence-based interventions will be essential to translating these findings into action, ultimately contributing to healthier schools, higher-quality instruction, and greater teacher satisfaction worldwide.

## Figures and Tables

**Table 1:** Theoretical explanations for potential moderators of the self-efficacy–job satisfaction relationship.

Moderator Category	Specific Moderator	Theoretical Mechanism Explanation
Teacher-level characteristics	School level at which teachers work	Teachers across elementary, middle, and high schools face distinct classroom challenges and student needs. According to social cognitive theory, these contextual differences shape the development and application of self-efficacy, thus influencing job satisfaction.
	Subject that teachers teach	Different teaching subjects vary in cognitive demands, classroom control, and emotional labor. These subject-specific challenges influence teachers’ perceived competence and task mastery, which directly affect self-efficacy and job satisfaction.
Country-level characteristics	English native-speaking countries vs. non-English native-speaking countries	Language and cultural context shape how self-efficacy is expressed and evaluated. Standardized assessments and policy environments in English-speaking countries may constrain perceived autonomy and efficacy.
	Developed vs. developing countries	In developed countries, better infrastructure, teacher training, and institutional support facilitate the development of self-efficacy. In contrast, systemic barriers in developing countries may hinder efficacy and reduce job satisfaction.
	Southern Hemisphere vs. Northern Hemisphere	Educational systems in the Southern Hemisphere often import curricula from Northern contexts without adequate adaptation, leading to a poor fit with local needs. This may weaken teachers’ perceived control and efficacy, lowering satisfaction.

**Table 2:** General characteristics of selected studies.

#	Study	r	N	Country	Type of Article	School Level	Subject Taught	Hemisphere	Language	Development Status
1	Akomolafe & Ogunmakin (2014) [103]	0.26	398	Nigeria	Journal	Pre-high school	All	North	Non-English	Developing country
2	Aldridge & Fraser (2016) [80]	0.71	781	Australia	Journal	After-high school	All	South	English	Developed country
3	Avanzi et al. (2013) [104]	0.35	348	Italy	Journal	Pre-high school	All	North	Non-English	Developed country
4	Barouch-Gilbert et al. (2014) [87]	0.29	75	Dominican Republic	Journal	Pre-high school	All	North	Non-English	Developing country
5	Blackburn et al. (2017) [81]	0.55	105	U.S.	Journal	After-high school	Single	North	English	Developed country
6	Briones et al. (2010) [105]	0.26	68	Spain	Journal	Pre-high school	All	North	Non-English	Developed country
7	Burić & Moè (2020) [106]	0.46	536	Croatia	Journal	After-high school	All	North	Non-English	Developing country
8	Çevik (2017) [107]	0.33	358	Turkey	Journal	After-high school	All	North	Non-English	Developing country
9	Canrinus et al. (2012) [108]	0.21	5575	Netherlands	Journal	Pre-high school	All	North	Non-English	Developed country
10	Capone & Petrillo (2020) [109]	0.52	285	Italy	Journal	After-high school	All	North	Non-English	Developed country
11	Caprara et al. (2006) [110]	0.53	2184	Italy	Journal	Pre-high school	All	North	Non-English	Developed country
12	Capri & Guler (2018) [111]	0.27	452	Turkey	Journal	Pre-high school	All	North	Non-English	Developing country
13	Cayupe et al. (2023) [112]	0.42	300	Peru	Journal	Pre-high school	All	South	Non-English	Developing country
14	Demir (2020) [113]	0.25	321	Turkey	Journal	Pre-high school	All	North	Non-English	Developing country
15	Demirdag (2015) [83]	−0.01	208	U.S.	Journal	Pre-high school	All	North	English	Developed country
16	Didonna (2018) [114]	0.26	122	U.S.	Dissertation	Pre-high school	All	North	English	Developed country
17	Fathi & Savadi Rostami (2018) [115]	0.55	312	Iran	Journal	After-high school	Single	North	Non-English	Developing country
18	Høigaard et al. (2012) [116]	0.30	192	Norway	Journal	After-high school	All	North	Non-English	Developed country
19	Karabiyik & Korumaz (2014) [79]	0.27	83	Turkey	Journal	Pre-high school	All	North	Non-English	Developing country
20	Kennedy (2014) [117]	0.35	110	U.S.	Dissertation	Pre-high school	All	North	English	Developed country
21	Kusumaninggati et al. (2018) [118]	0.72	172	Indonesia	Journal	After-high school	All	South	Non-English	Developing country
22	Lent et al. (2011) [119]	0.37	235	Italy	Journal	After-high school	All	North	Non-English	Developed country
23	Li et al. (2017) [120]	0.42	352	China	Journal	Pre-high school	All	North	Non-English	Developing country
24	Moè et al. (2010) [86]	0.33	399	Italy	Journal	After-high school	All	North	Non-English	Developed country
25	Ortan et al. (2021) [12]	0.42	658	Romania	Journal	Pre-high school	All	North	Non-English	Developing country
26	Renbarger & Davis (2019) [121]	0.29	226	U.S.	Journal	Pre-high school	All	North	English	Developed country
27	Rincon (2018) [122]	0.54	14	U.S.	Dissertation	Pre-high school	All	North	English	Developed country
29	Reilly et al. (2014) [82]	−0.04	121	Ireland	Journal	Pre-high school	All	North	English	Developed country
29	Safari et al. (2020) [123]	0.64	212	Iran	Journal	After-high school	Single	North	Non-English	Developing country
30	Skaalvik & Skaalvik (2014) [124]	0.41	2569	Norway	Journal	Pre-high school	All	North	Non-English	Developed country
31	Skaalvik & Skaalvik (2017) [125]	0.33	760	Norway	Journal	Pre-high school	All	North	Non-English	Developed country
32	Somech & Drach-Zahavy (2000) [126]	0.16	251	Isreal	Journal	Pre-high school	All	North	Non-English	Developed country
33	Song et al. (2020) [127]	0.30	1525	China	Journal	Pre-high school	All	North	Non-English	Developing country
34	Stephanou et al. (2013) [128]	0.77	268	Greece	Journal	Pre-high school	All	North	Non-English	Developed country
35	Troesch & Bauer (2017) [129]	0.36	193	Switzerland	Journal	After-high school	All	North	Non-English	Developed country
36	Viel-Ruma et al. (2010) [84]	0.29	70	U.S.	Journal	After-high school	All	North	English	Developed country
37	Watts (2013) [130]	0.38	171	U.S.	Dissertation	Pre-high school	All	North	English	Developed country
38	Yıldırım (2015) [131]	0.30	306	Turkey	Journal	After-high school	Single	North	Non-English	Developing country
39	Yıldız & Şimşek (2016) [132]	0.55	252	Turkey	Journal	After-high school	All	North	Non-English	Developing country

**Table 3:** Overall effect sizes.

	k	Effect Size	SE	Variance	95% CI		Test of Heterogeneity		
					LL	UL	Q-Value	df (Q)	I^2^
Fixed	39	0.39	0.01	0.00	0.36	0.40	802.07 ***	38	95.26
Random	39	0.41	0.04	0.00	0.34	0.49			

Note. k: number of effect sizes; CI: confidence interval; *** *p* < 0.001.

**Table 4:** Results of the moderator analysis.

Moderator	k	Effect Size	SE	95% CL		Z-Value	*p*-Value	Q-Value
				LL	UL			
School level								7.09 *
Pre-high school	24	0.34	0.04	0.26	0.42	8.20	0.00	
After-high school	15	0.52	0.05	0.42	0.63	9.71	0.00	
English-speaking country								0.70
Yes	10	0.36	0.09	0.19	0.54	4.02	0.00	
No	29	0.43	0.04	0.36	0.50	11.63	0.00	
Developed country								0.44
Yes	23	0.39	0.05	0.29	0.49	7.70	0.00	
No	16	0.44	0.05	0.35	0.53	9.27	0.00	
Subject								2.30
Single subject	4	0.57	0.10	0.38	0.76	5.94	0.00	
All subjects	35	0.39	0.04	0.32	0.46	10.57	0.00	
Hemisphere								9.03 *
Northern	35	0.38	0.03	0.32	0.45	11.61	0.00	
Southern	4	0.75	0.15	0.45	1.04	4.94	0.00	

Note. k: numbers of effect sizes; * *p* < 0.01.

## Data Availability

This study is a meta-analysis based on previously published articles. All data analyzed in this study were extracted from publicly available sources. No new data were collected by the authors.
